# Flavopiridol causes cell cycle inhibition and demonstrates anti-cancer activity in anaplastic thyroid cancer models

**DOI:** 10.1371/journal.pone.0239315

**Published:** 2020-09-24

**Authors:** Nicole Pinto, Stephenie D. Prokopec, Farhad Ghasemi, Jalna Meens, Kara M. Ruicci, Imran M. Khan, Neil Mundi, Krupal Patel, Myung W. Han, John Yoo, Kevin Fung, Danielle MacNeil, Joe S. Mymryk, Alessandro Datti, John W. Barrett, Paul C. Boutros, Laurie Ailles, Anthony C. Nichols

**Affiliations:** 1 Department of Otolaryngology–Head and Neck Surgery, Western University, London, Ontario, Canada; 2 Department of Anatomy and Cell Biology, Western University, London, Ontario, Canada; 3 Ontario Institute for Cancer Research, Toronto, Ontario, Canada; 4 Princess Margaret Cancer Centre, University Health Network, Toronto, Ontario, Canada; 5 Department of Oncology, Western University, London, Ontario, Canada; 6 Department of Microbiology and Immunology, Western University, London, Ontario, Canada; 7 Network Biology Collaborative Centre, Lunenfeld-Tanenbaum Research Institute, Mount Sinai Hospital, Toronto, Ontario, Canada; 8 Department of Agricultural, Food and Environmental Sciences, University of Perugia, Perugia, Italy; 9 Department of Medical Biophysics, University of Toronto, Toronto, Ontario, Canada; 10 Department of Pharmacology & Toxicology, University of Toronto, Toronto, Ontario, Canada; 11 Department of Human Genetics, University of California, Los Angeles, CA, United States of America; 12 Department of Urology, University of California, Los Angeles, CA, United States of America; 13 Broad Stem Cell Research Center, University of California, Los Angeles, CA, United States of America; 14 Institute for Precision Health, University of California, Los Angeles, CA, United States of America; 15 Jonsson Comprehensive Cancer Center, University of California, Los Angeles, CA, United States of America; Columbia University, UNITED STATES

## Abstract

Anaplastic thyroid cancer (ATC) is a rare, but nearly uniformly fatal disease that is typically resistant to chemotherapy and radiation. Alternative strategies to target this cancer at a molecular level are necessary in order to improve dismal outcomes for ATC patients. We examined the effects of flavopiridol, a CDK inhibitor, in a panel of ATC cell lines. When cell lines were treated over a ten-point concentration range, CAL62, KMH2 and BHT-101 cell lines had a sub micromolar half-maximal inhibitory concentration, while no effect was seen in the non-cancerous cell line IMR-90. Flavopiridol treatment resulted in decreased levels of the cell cycle proteins CDK9 and MCL1, and induced cell cycle arrest. Flavopiridol also decreased the *in vitro* ability of ATC cells to form colonies and impeded migration using a transwell migration assay. *In vivo*, flavopiridol decreased tumor weight and tumor volume over time in a patient-derived xenograft model of ATC. Given the observed *in vitro* and *in vivo* activity, flavopiridol warrants further investigation for treatment of ATC.

## Introduction

Anaplastic thyroid cancer (ATC) is a rare form of undifferentiated thyroid cancer which progresses rapidly and is almost universally fatal [1–3]. While well-differentiated thyroid cancers are typically indolent with cure rates in excess of 90% of cases, those diagnosed with ATC experience a median survival of approximately 6 months following diagnosis [4–7]. Even with the use of conventional therapies such as surgical resection, chemotherapy and radiation, improvements in overall patient survival for those diagnosed with ATC have not changed in the past decade, remaining at a dismal mortality rate of close to 100% [1,8].

The failure to increase survival rates of those suffering from ATC using the aforementioned treatment strategies has shifted current efforts to determine which molecular targets are “actionable” and can be used to halt the otherwise rapid disease progression. The use of small-molecule kinase inhibitors to target key proteins has become an attractive strategy across many cancer types including a number of prospective targeted therapies which have been identified for inhibition of various putative targets in ATC including sorafenib (VEGFR), axitinib (VEGFR), vemurafenib and dabrafenib (BRAF/MEK), imatinib (PDGFR), and selumetinib (MEK 1 and 2) [9]. Similarly, cell cycle inhibitors, specifically cyclin-dependent kinase (CDK) inhibitors, are under active investigation in a number of other cancer types, including breast cancer and non-small cell lung cancer [10–12].

In this study we focus on the small-molecule inhibitor flavopiridol, a reported pan-CDK inhibitor. Treatment with flavopiridol in acute myeloid leukemia has been shown to result in the inhibition of CDKs, specifically CDK9 resulting in down-regulation of MCL1 protein expression and the induction of apoptosis [13,14]. Further, flavopiridol has been described in the literature to be a potent cell cycle inhibitor with the potential to be used as a radiosensitizer in some cancer types, including esophageal and ovarian cancer cell lines and in 2014 this compound was granted orphan drug status by the FDA for the treatment of acute myeloid leukemia (AML), thus making this compound an interesting anti-cancer agent [15–17].

In the present study, we examine the activity of flavopiridol in ATC. We observed that flavopiridol is a potent inhibitory compound across our ATC cell lines with nanomolar inhibitory concentrations. We demonstrate that flavopiridol exhibits efficacy in ATC models by reducing *in vitro* cell proliferation, colony formation and migration, decreasing MCL1 signaling and inducing cell cycle arrest. Finally, we demonstrate that flavopiridol has significant activity *in vivo* in a patient-derived xenograft model of ATC.

## Materials and methods

### Cell lines and culture conditions

The ATC cell line KMH2 was purchased from the Japanese collection of Research of Bioresources Cell Bank (JCRB; Osaka, Japan). BHT-101 and CAL62 cell lines were both purchased from the DSMZ Cell Bank (DSMZ Cell Bank, Germany, EU). All cell lines were cultured in DMEM supplemented with 10% heat inactivated FBS (GIBCO; Thermo Fisher Scientific, Carlsbad, CA, USA), penicillin (100 μg/mL) and streptomycin (100 μg/mL) (Invitrogen; Thermo Fisher Scientific, Carlsbad, CA, USA). Short tandem repeat (STR) profiling of all cell lines was completed as described by Pinto et al. 2018 [18]. The lung fibroblast cell line IMR-90 was purchased from ATCC (ATCC, Manassas, VA, USA) and the media used for culture was EMEM supplemented with 10% FBS (GIBCO), penicillin (100 μg/mL) and streptomycin (100 μg/mL) (Invitrogen). All cell lines were incubated at 37ºC and 5% CO_2_.

### Drug treatment

Flavopiridol was purchased from Selleckchem (Burlington, ON, Canada; Cat no. S1230). The drug was dissolved in dimethyl sulfoxide (DMSO; Sigma-Aldrich, Oakville, ON, Canada) and prepared as a 10mM stock solution. Subsequently, serial dilutions were performed for the initial drug testing (final concentrations of 0.0075–4μM).

The normal lung fibroblast cell line (IMR-90) and ATC cell lines (KMH2, BHT-101 and CAL62) were seeded into 96-well plates at a density of 2,400 cells per well and incubated at 37°C and 5% CO_2_. After 24hrs, cells were treated with an extended dose range of flavopiridol concentrations from 0.06 to 32μM. After 72hrs, plates were read by incubating cells with PrestoBlue (Thermo Fisher Scientific) for 1hr at 37°C. After incubation with PrestoBlue (Thermo Fisher Scientific), fluorescence readings were completed using a BioTek Synergy Microplate Reader with 560nm excitation and 590nm emission wavelengths. To generate dose-response curves, raw fluorescence data was loaded into Prism 7 GraphPad Software, where the values were then normalized to the untreated control samples. To determine the IC_50_, the normalized relative fluorescence units (RFU) of the flavopiridol-treated samples were calculated as a percentage of the mean RFU of the untreated samples. Drug concentrations were then transformed to a logarithmic scale and IC_50_ values were calculated using non-linear regression (curve fit).

### Immunoblotting

Cells were seeded at a density of 200,000 cells/well in 6-well plates and incubated overnight. After 24hrs, cells were treated with flavopiridol (0.25, 0.50, 1.0, 2.0 and 4.0 μM) and incubated for either 24 or 48hrs. Cells were lysed, and a Bradford assay was performed to determine the protein concentration of the whole cell lysates using the Bio-rad Protein Assay Dye Reagent (Bio-Rad, Mississauga, ON, Canada). NuPAGE® Novex® 4–12% Bis-Tris Gel (Thermo Fisher Scientific) was used for protein separation with 20 μg of total protein loaded per well and run at 200V (1 hour). Novex® Sharp Standard protein ladder was used to determine the mass of products. A PVDF blotting membrane (GE Healthcare, Mississauga, ON, Canada) was used for protein transfer at 14V (1 hour). Membranes were blocked using 5% bovine serum albumin (Sigma-Aldrich, Oakville, ON, Canada). Primary antibodies were prepared according to the manufacturer and incubated overnight at 4°C with constant, mild agitation. Antibodies used included CDK9 (Cat no. 2316), phospho CDK9 (Thr186) (Cat no. 2549), MCL1 (Cat no. 39224) and *α*-tubulin (Cat no. 2125) obtained from Cell Signaling Technology (Whitby, ON, Canada). Secondary antibodies were diluted 1:5000. Detection of target proteins was performed using Luminata Forte Western HRP substrate (EMD Millipore, Burlington, MA, USA). α-tubulin was used as a loading control.

### Clonogenic assay

CAL62 and BHT-101 cells were seeded into 6-well plates at a density of 1,000 cells per well. After 24hrs, cells were treated with 60 and 125nM flavopiridol and incubated at 37°C for 7 days based on colony formation of the control-treated wells. Cells were washed with PBS and fixed with 100% chilled methanol for 15 minutes. Following fixation, cells were stained with 0.5% crystal violet in 10% methanol/1x PBS for 10 minutes. Brightfield microscopy was used to quantify colony formation, with a colony defined as being ≥ 50 cells. For quantification, three representative fields per well were counted for the number of colonies and this was completed for both the control and the varying concentrations of flavopiridol tested.

### Cell migration assay

Cell migration for CAL62, BHT-101 and KMH2 cell lines was assessed using a 24-well cell transwell migration plate and fluorometric analysis (Cat no. CBA-100; Cell Biolabs Inc, San Diego, CA, USA). Initial cell suspensions (5x10^4^ cells per well) were placed into the upper chambers containing 60 nM and 125 nM concentrations of flavopiridol in serum-free media, with two biological replicates. Cells were incubated at 37°C in order to allow migratory cells to migrate through the polycarbonate membrane to the bottom of the chamber. Cells that stayed in the upper chamber were considered to be non-migratory. Migratory cells were then dissociated from the membrane of the chamber using the Cell Detachment Buffer provided in the cell migration plate kit. Cells which migrated through were lysed and quantified using CyQuant GR Fluorescent Dye provided in the same kit. Fluorescence readings were completed using a BioTek Synergy Microplate Reader.

### Cell cycle analysis

KMH2, CAL62 and BHT-101 cells were treated with either DMSO-only or 125nM flavopiridol for 24hrs, with 3 biological replicates each. Bromo-deoxyuridine (BrdU; Cat no. RPN201, GE Healthcare) was added to cell media and incubated in culture for 2hrs prior to collection. Cell were trypsinized, collected and washed 3 times with PBS prior to using 95% freezer-chilled ethanol to fix cells. Cells were permeabilized using 2N HCl/0.5% Triton X-100, followed by 0.1 M NaB_4_O_7_. Primary (mouse anti-BrdU primary antibody, 1:50; BD Biosciences lot. 347580) and secondary (FITC-conjugated rabbit anti-mouse secondary antibody, 1:25; Cat no. FI-2000, Vector Laboratories, Burlingame, CA, USA) antibodies were added and incubated for 30 minutes for each step. Cell pellets were resuspended in propidium iodide (PI; 10 mg/ml) and RNase A (0.25 mg/ml; Cat no. RNA675, Bioshop Canada Inc., Burlington, ON, Canada) and incubated overnight at 4°C. The following day, cell cycle analysis using flow cytometry was performed (Beckman Coulter Inc, Cytomics FC500).

### RNA interference and drug treatment

KMH2 and CAL62 cells were seeded into 6-well plates at a confluency of ~40% at transfection. Lipofectamine® RNAiMAX Reagent (Cat no. 13778030, Thermo Fisher Scientific) was used for transfection purposes. ON-TARGETplus CDK9 siRNA (Cat no. L-003243- 00–0002, Dharmacon, Lafayette, CO, USA) and scrambled negative siRNA transfection control (siCT) (Cat no. 4390843, Thermo Fisher Scientific) were used for transfections in Opti-MEM at a concentration of 10 μM. Media was replaced after 24hrs. Cells were incubated for 72hrs and proliferation was measured every 24hrs by the addition of PrestoBlue and measuring the resulting fluorescence values, which were then normalized. Immunoblotting was used to determine levels of knockdown for each cell line relative to the siCT.

Both siCT and siCDK9 cells for KMH2 and CAL62 cell lines were seeded into 96-well plates at a density of 2,400 cells per well and incubated at 37°C and 5% CO_2_. After 24hrs, cells were treated with an extended dose range of flavopiridol concentrations from 0.06 to 4.0 μM. After 72hrs, plates were read by incubating cells with PrestoBlue (Thermo Fisher Scientific) for 1hr at 37°C. After incubation with PrestoBlue (Thermo Fisher Scientific), fluorescence readings were completed using a BioTek Synergy Microplate Reader with 560nm excitation and 590nm emission wavelengths. To generate dose-response curves, raw fluorescence data was loaded into Prism 7 GraphPad Software, where the values were then normalized to the untreated control samples. To determine the IC_50_, the normalized relative fluorescence units (RFU) of the flavopiridol-treated samples were calculated as a percentage of the mean RFU of the untreated samples. Drug concentrations were then transformed to a logarithmic scale and IC_50_ values were calculated using non-linear regression (curve fit).

### Study approval

Informed consent was obtained from the participant and a fresh surgical ATC specimen was received from the consenting patient with diagnosed ATC who underwent surgery at the London Health Sciences Centre under a Research Ethics Board approved protocol (REB# 108920). Mice used in this study were in accordance with AUP 1542, which was approved by the University Health Network Animal Care Committee and the CCAC regulations.

### Establishment of a patient-derived xenograft model

Informed consent was obtained from the participant and a fresh tumor sample was obtained from the tumor tissue of a 70-year old female with confirmed ATC (tumor (T) category 4, lymph node (N) category 1 and with distant metastasis to the lung (M) category 1 –T4N1M1) who underwent surgery at the London Health Sciences Centre. The ATC sample was received 15 minutes after an open biopsy and was kept at 4°C in PBS until engraftment 24hrs post biopsy. The tumor was divided into ~1mm^3^ pieces and further used for implanting subcutaneously into NOD/SCID/IL2R2γ-/- (NSG) male mice (flank region). Mice were sacrificed using CO_2_ asphyxiation when tumors reached a size of 1–1.5 cm and tumors were then dissected. Upon dissection, tumors were dissociated in culture medium (containing DNASE 1 and collagenase/hyaluronidase) [19]. Dissociated tumors were then passaged subcutaneously with five mice per treatment (minimum of 100,000 cells/mouse) in 1:1 Matrigel/PBS to generate the PDX models [19]. Once tumors were palpable in the mice, calipers were used to take periodic measurements.

### PDX drug treatment and specimen collection

Five xenografts were established per treatment and randomized to either flavopiridol (7.5mg/kg/day) by intraperitoneal injection or a vehicle control (water). Mice were treated for a period of 17 days (five days on with two days break in between) with the treatment beginning at 14 days post-injection until the controls reached the endpoint at day 35. Mice in the control-arm were maintained until an alternative humane endpoint was reached or when tumors reached a max size of 1.5 cm in diameter (as stated in the animal protocol). Overall health was observed on a daily basis and tumor size and weight were evaluated every 2–4 days. Tumor volume was calculated using the formula: [length x (width)^2^] x 0.52 [19]. For each experimental group, mean tumour weight was calculated ± standard deviation (grams; g) and an unpaired, two-tail *t*-test was performed to determine statistical differences. Mice were sacrificed using CO_2_ asphyxiation and tumors were then dissected and were stored in formalin followed by formalin-fixed paraffin embedding (FFPE) and immunohistochemistry (IHC).

### Immunohistochemistry

IHC staining was carried out in collaboration with the Department of Pathology & Laboratory Medicine and the Molecular Pathology Core Facility (Western University) where 4-μM sections were cut and tested for expression of thyroid-related markers. Primary antibodies used for staining were used according to the manufacturer’s instructions: CDK9 (Cell Signaling Technology; 2546S), MCL1 (Cell Signaling Technology; 39224) and Ki67 (Cell Signaling Technology; 9027S). Slides were scanned using an Aperio ScanScope slide scanner. Images were imported into Fiji (ImageJ) software [20] and Ki67 staining was assessed and the percentage of stained cells were calculated.

### Statistical analysis

A Student’s unpaired, two-tail *t*-test was performed where appropriate using Prism® 7 Graphpad Software Macintosh Version (by Software MacKiev © 1994–2014 GraphPad Software, Inc). *P* values < 0.05 were considered statistically significant. *P* values are defined as ns *p* > 0.05, * represents *p* < 0.05, ** represents *p* < 0.01, and *** represents *p* < 0.001.

## Results

### Flavopiridol inhibits growth, migration and colony formation in ATC cell lines

**Fig 1:**
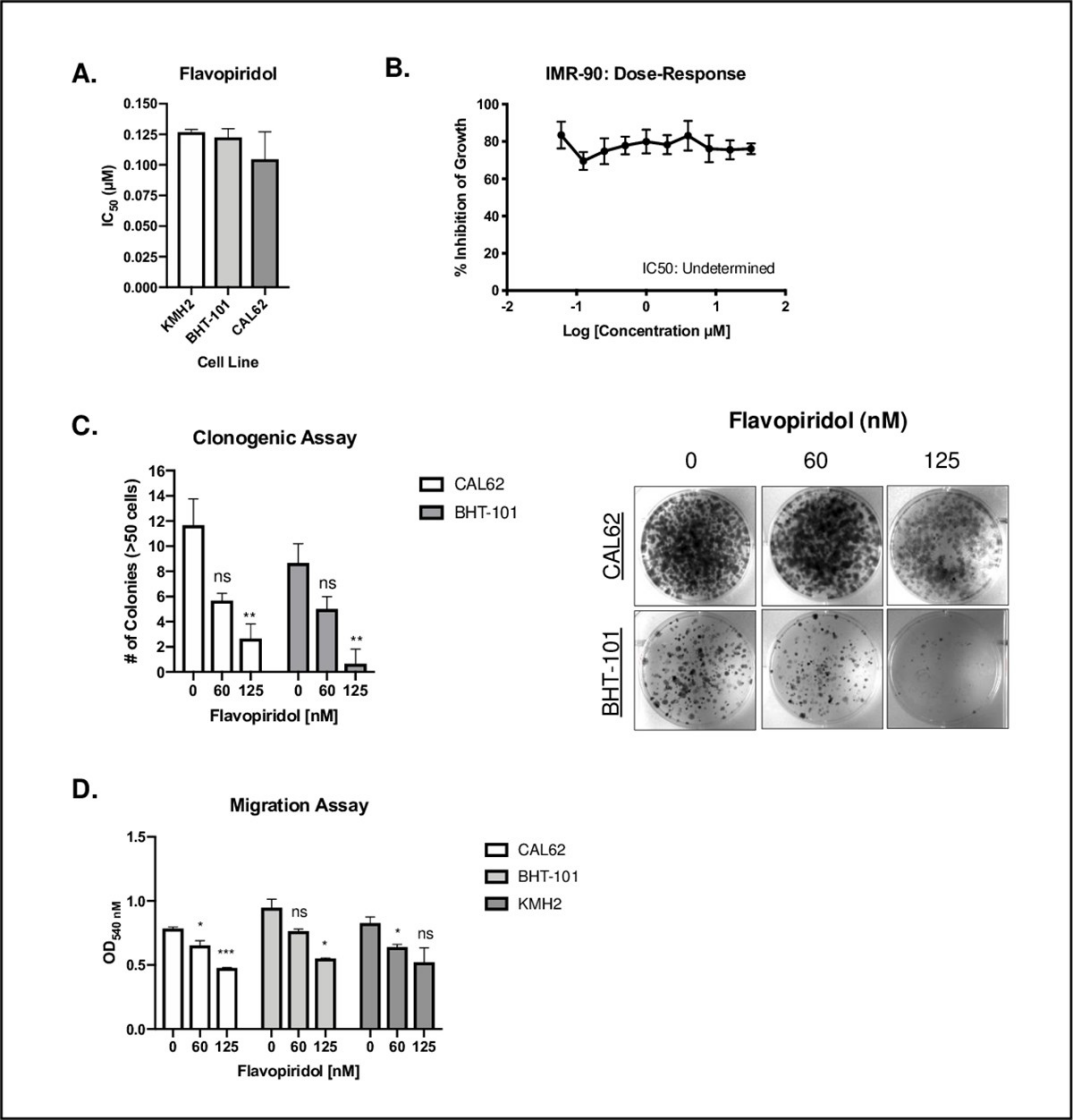
Flavopiridol inhibits cell growth, clonogenic capabilities and migration in ATC cell lines. **(A)** Flavopiridol was tested against 3 ATC cell lines, with 2 biological replicates per cell line. Cells were treated with flavopiridol for 48hrs and plate readings were completed after incubation with Presto Blue. Mean IC_50_ values are shown ± standard deviation (indicated by the error bar). **(B)** Dose-response curve demonstrating the sensitivity of the lung fibroblast cell line, IMR-90, over 10 concentrations (0.06 to 32μM) of flavopiridol with 2 biological replicates. Mean values for each concentration are shown ± standard deviation as indicated by the error bars. **(C)** Clonogenic assays comparing untreated and treated (60 and 125nM flavopiridol) ATC cell lines (CAL62 and BHT-101) after 7 days. Colonies were counted and graphed for 3 representative fields from each well (3 technical replicates per cell line as indicated by error bars showing the standard deviation). The number of colonies were compared to the untreated control for each cell line. **(D)** Cell migration was quantified using fluorometric analysis. Cells (CAL62, BHT-101 and KMH2) were seeded into the top chamber containing serum-free media at a density of 5x10^4^ cells per chamber with either 60 or 125 nM flavopiridol (2 biological replicates per concentration tested as indicated by the error bars showing the standard deviation). Each chamber was then placed into a well containing media with serum. Cells which migrated through to serum-containing media after 24 hours were quantified using fluorometric detection as described using the standard protocol provided by the manufacturer. Fluorescence readings were completed using a microplate reader. * represents *p* < 0.05, ** represents *p* < 0.01, *** represents *p* < 0.001, ns = not significant, unpaired Student’s, two-tail *t-*test.

Cell lines were treated with increasing concentrations of flavopiridol to determine the half maximal inhibitory concentration (IC_50_) values for each. All three ATC cell lines (KMH2, BHT-101 and CAL62) had an IC_50_ value below 0.13μM when treated with flavopiridol (Fig 1A). KMH2 had an IC_50_ of 0.13 ± 0.002 μM, BHT-101 had an IC_50_ of 0.12 ± 0.007 μM and CAL62 had an IC_50_ of 0.10 ± 0.02 μM (Fig 1A). To further evaluate whether flavopiridol has preferential activity in cancer cells, we treated the lung fibroblast cell line IMR-90 as a normal control. When treated with a concentration range from 0.06 to 32μM, the IMR-90 cell line did not appear to reach an IC_50_ (Fig 1B).

As flavopiridol has been reported to be cytostatic [21], we next assessed the clonogenic capabilities of CAL62 and BHT-101 cells when treated with 60 nM and 125 nM concentrations of flavopiridol (concentrations based on IC_50_ values for cell lines). For both cell lines, treatment with flavopiridol (125 nM) resulted in a significant decrease in the ability of cells to form colonies compared to the untreated control (*p* = 0.005 and *p* = 0.004 for BHT-101 and CAL62, respectively). This effect was not significant at a concentration of 60 nM (*p* = 0.09 and *p* = 0.06 for BHT-101 and CAL62, respectively) (Fig 1C).

Flavopiridol has also been reported to inhibit cell migration by altering cell adhesion leading to suppression of migration [22]. Cell migration was quantified using a transwell migration assay and fluorometric analysis, where CAL62, BHT-101 and KMH2 cells were seeded into serum free media containing 60 or 125 nM flavopiridol. With flavopiridol treatment, there was a significant decrease in the number of cells migrating through the polycarbonate membrane as measured using fluorescence when cells were detached, lysed and quantified. When quantified using fluorescence, the OD_540_ of untreated cells was 0.78 ± 0.01 versus 0.65 ± 0.03 (*p* = 0.04) for CAL62 cells treated with 60 nM flavopiridol (Fig 1D). When CAL62 cells were treated with 125 nM flavopiridol, the OD_540_ further decreased to 0.48 ± 0.003 (*p* = 0.0007) (Fig 1D). Likewise, when BHT-101 cells were treated with flavopiridol, there was a significant decrease in OD_540_ from 0.95 ± 0.07 in the untreated versus 0.55 ± 0.003 (*p* = 0.01) in the 125 nM flavopiridol treated cells (Fig 1D). Similar to both CAL62 and BHT-101 cell lines, we observed a significant decrease in OD_540_ when KMH2 cells were treated with 60 nM flavopiridol (0.64 ± 0.02) compared to untreated (0.83 ± 0.05) (*p* = 0.04) (Fig 1D).

### Flavopiridol downregulates cell cycle proteins and induces cell cycle arrest and apoptosis

**Fig 2:**
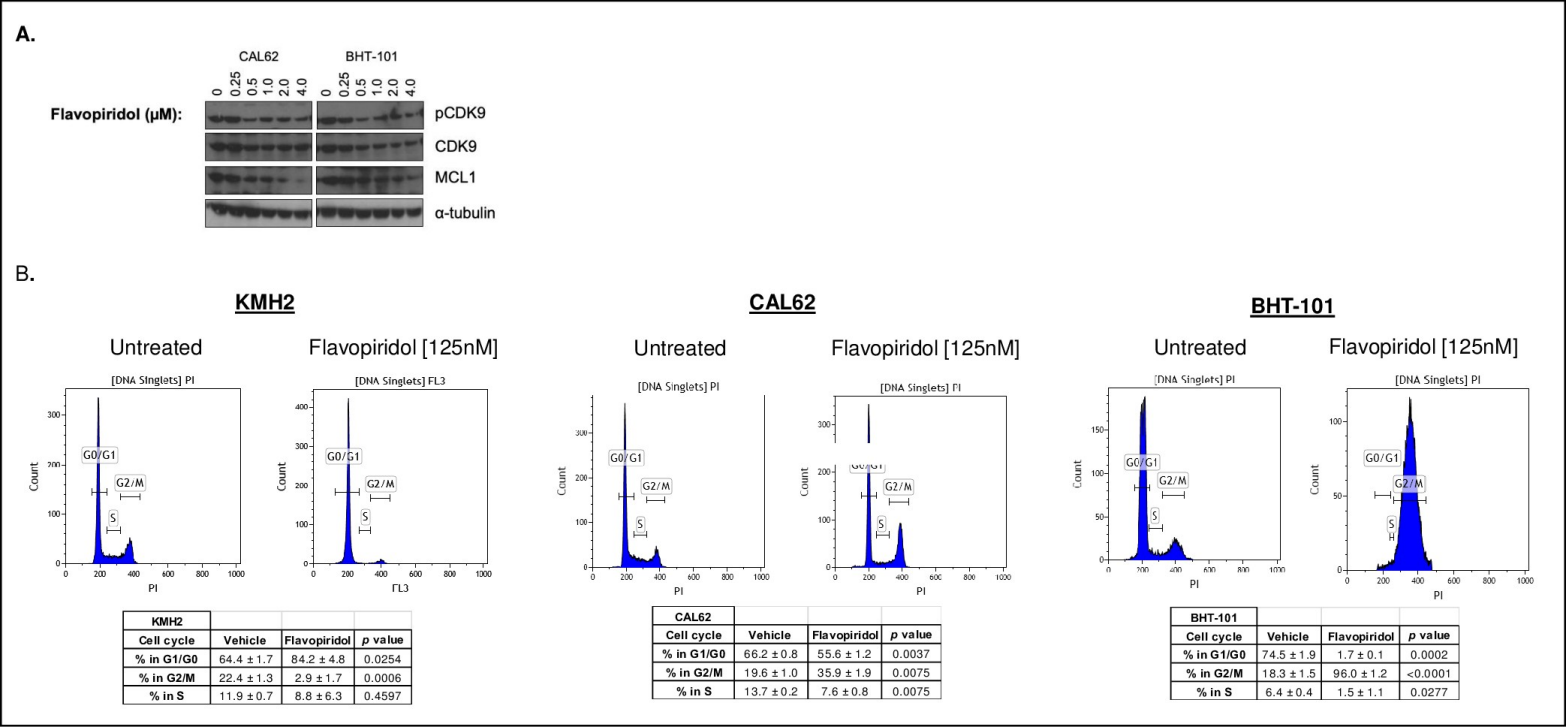
Flavopiridol treatment results in cell cycle arrest *in vitro*. **(A)** Immunoblot of CAL62 and BHT-101 cells treated with flavopiridol for 24hrs. Cells were collected after drug treatment and whole cell lysates were prepared with 20 μg total protein loaded per well. **(B)** KMH2, CAL62 and BHT-101 cells were exposed to the vehicle control or flavopiridol (125nM) for 24hrs, with 3 biological replicates per cell line, prior to BrdU incorporation and labeling with propidium iodide. A minimum of 10,000 events was counted and the proportion of cells present in each phase of the cell cycle ± standard deviation is shown.

To assess whether treatment of ATC cell lines with flavopiridol affected the steady state protein levels or phosphorylation status of its reported target (CDK9), immunoblotting was carried out. Treatment with flavopiridol for 24hrs in CAL62 and BHT-101 cells resulted in a decrease in phosphorylation of CDK9 (pCDK9) (Fig 2A). We also assessed the changes in the anti-apoptotic regulator MCL1, as previous studies have demonstrated a depletion of the anti-apoptotic protein MCL1 in response to flavopiridol drug treatment [23]. With increasing concentrations of flavopiridol drug treatment, a corresponding reduction of MCL1 expression was also observed (Fig 2A).

We next used flow cytometry to assess the changes in cell cycle upon exposure to flavopiridol (125nM) for 24hrs. Flavopiridol has previously been described as a cytostatic agent, with the potential to induce apoptosis at higher concentrations [24]. When the KMH2 cell line was treated with 125 nM flavopiridol for 24hrs, a greater proportion of cells appeared to accumulate in the G1/G0 phase (84.2 ± 4.8%) compared to the vehicle control cells (64.4 ± 1.7%) (*p* = 0.03) (Fig 2B). When the CAL62 cell lines were treated with 125 nM flavopiridol, a greater proportion of cells appeared to accumulate in the G2/M phase (35.9 ± 1.9%) compared to the vehicle control cells (19.6 ± 1.0%) (*p* = 0.01) (Fig 2B). Likewise, BHT-101 cells also demonstrated cell cycle arrest in the G2/M phase of the cell with 96.0 ± 1.2% of cells were arrested in the G2/M phase compared to 18.3 ± 1.5% in the vehicle-control group (*p* < 0.0001) (Fig 2B).

### siRNA knockdown of CDK9 results in variable impairment of proliferation and does not result in resistance to flavopiridol in ATC cell lines

**Fig 3:**
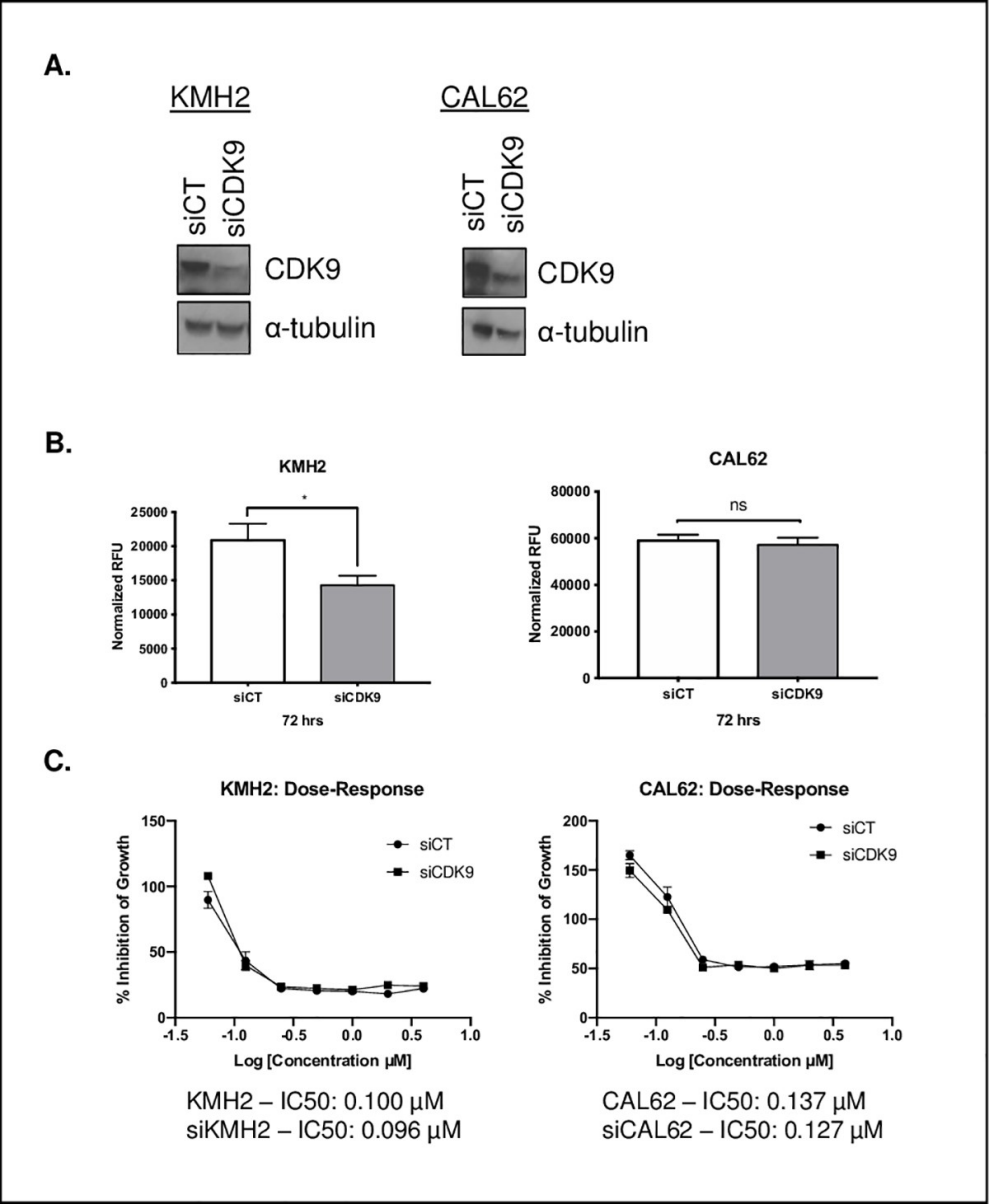
siRNA knockdown of CDK9 impairs ATC cell proliferation. **(A)** Immunoblot (20 μg total protein) of CDK9 expression in KMH2 and CAL62 cell lines showing the siCT relative to the siCDK9 knockdown. **(B)** Changes in cell proliferation with siRNA-mediated knockdown with Lipofectamine® RNAiMAX Reagent of CDK9 (siCDK9; 10 μM) relative to the scrambled control (siCT; 10 μM) in KMH2 and CAL62 cells (3 technical replicates as indicated by the error bars showing the standard deviation). * represents *p* < 0.05, ** represents *p* < 0.01, *** represents *p* < 0.001, ns = not significant, unpaired Student’s, two-tail *t-*test. **(C)** Dose-response curves of KMH2 and CAL62 cell lines comparing the siCT and siCDK9 cell lines treated with flavopiridol (0.06–4.00 μM) with their respective IC_50_ values (3 technical replicates per dose as indicated by the error bars showing the standard deviation).

Many kinase inhibitors are characterized as having off-target effects, making it unclear what their true anti-cancer mechanism is [25,26]. To assess whether specific CDK9 inhibition alone could result in an antiproliferative effect, we knocked down CDK9 (siCDK9) in CAL62 and KMH2 cell lines and compared it to a scrambled control. siRNA knockdown resulted in markedly lower CDK9 protein levels as expected (Fig 3A). Knockdown of CDK9 in the KMH2 cell line demonstrated a significant decrease in cell proliferation when measured at 72hrs (*p* = 0.01); however, knockdown of CDK9 in CAL62 did not significantly affect cell proliferation (*p* = 0.34) (Fig 3B). We then sought to determine whether knockdown of CDK9 as a target would result in an increase in resistance with a higher IC_50_ value of the siCDK9 cell line relative to the siCT cell line. Overall, for the KMH2 cell line, the siCT IC_50_ went from 0.100 μM to 0.096 μM with siCDK9 knockdown (Fig 3C). Likewise, for the CAL62 cell line, knockdown of CDK9 did not appear to make the cell line more resistant, with the IC_50_ in the siCT going from 0.137 μM to 0.127 μM in the siCDK9 cell line (Fig 3C).

### Flavopiridol inhibits tumor growth in a PDX model of ATC

**Fig 4:**
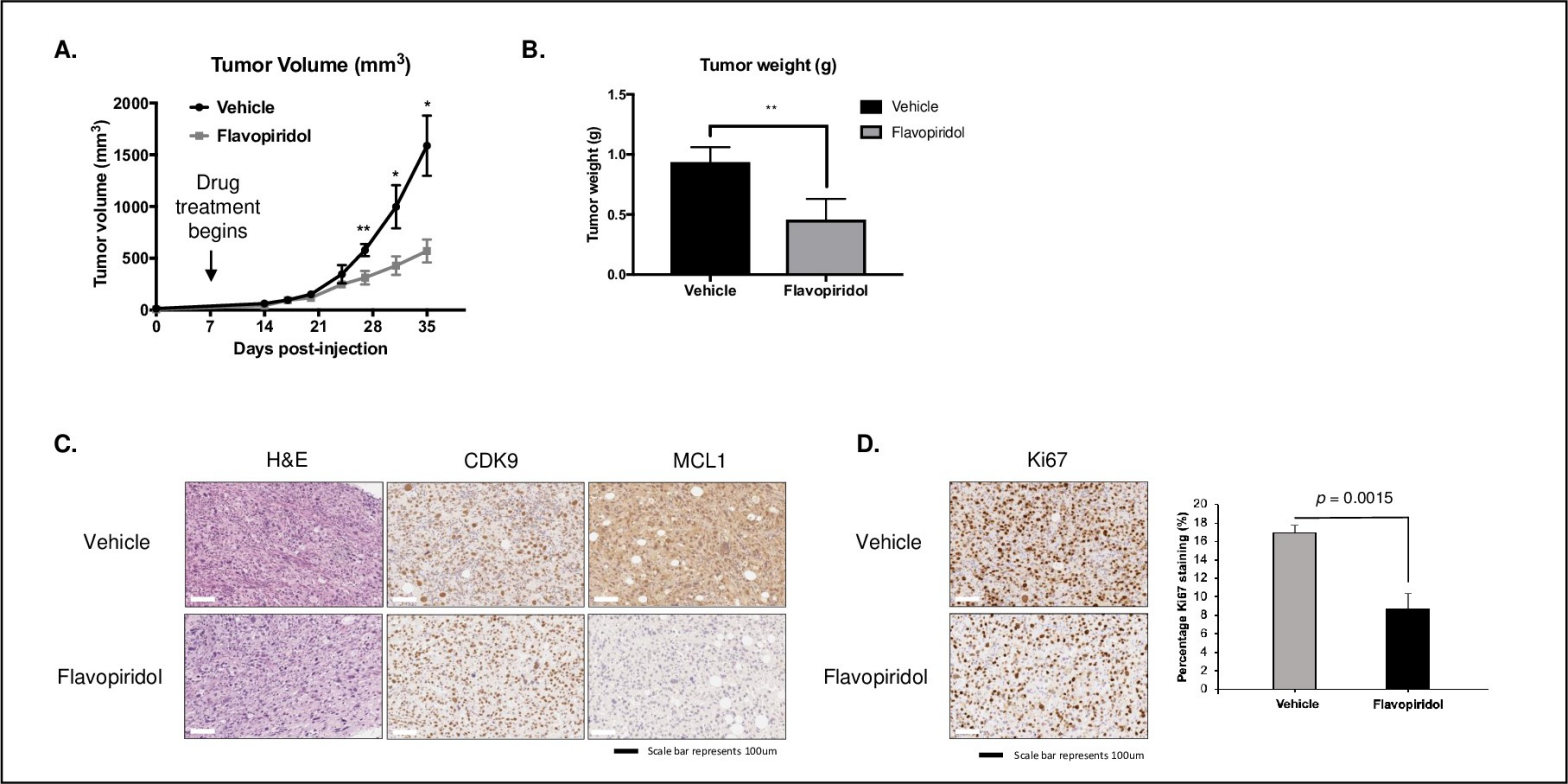
Flavopiridol inhibits tumor growth in a PDX model of ATC. **(A)** Primary tumor was obtained from a 70-year old female (with informed consent) with diagnosed ATC stage T4N1M1. Five xenografts (per treatment) were generated and randomized to either daily flavopiridol (7.5 mg/kg/day) by intraperitoneal injection or a vehicle control (water). Mice were treated with flavopiridol for a period of 17 days (five days on, two days break in between) with the treatment beginning at 14 days post injection until controls reached endpoint at day 35. Standard deviations are indicated by error bars at each timepoint. **(B)** Mice were evaluated for tumor weight (g) comparing the vehicle control group to the flavopiridol treated group at endpoint. Standard deviations indicated by error bars at each timepoint. * represents *p* < 0.05, ** represents *p* < 0.01, *** represents *p* < 0.001, ns = not significant, unpaired Student’s, two-tail *t-*test. **(C)** Representative IHC sections of the vehicle and flavopiridol-treated specimens demonstrating H&E staining in the PDX model of ATC in addition to protein expression of CDK9 and MCL1. **(D)** Representative IHC sections of the vehicle and flavopiridol-treated specimens demonstrating Ki67 protein expression in the PDX model of ATC. Standard deviations indicated by error bars.

A primary tumor from a 70-year old female patient (with informed consent) was used for the development of PDX models. The patient was diagnosed with ATC–tumor (T) category 4, lymph node (N) category 1 and with distant metastasis to the lung (M category 1). Two mice were initially excluded from this study due to low weight (one from the control group and one from the flavopiridol treatment group). Final data analysis was completed for the control group, which had an n = 4 and the flavopiridol treatment group (n = 2), with two mice dying prior to the endpoint of the experiment (4 and 8 days prior). Beyond day 27, the tumors in the flavopiridol treated PDX models were significantly smaller than those treated with placebo (Fig 4A). Furthermore, when we assessed tumor weight at the end of our study, we found that the vehicle control group had a mean tumor weight of 0.94 ± 0.13 g (mean tumor weight ± standard deviation g) and mice treated with flavopiridol showed a mean tumor weight of 0.46 ± 0.17 g (*p* = 0.02) (Fig 4B). After completion of the study, tumors were excised from the mouse models for FFPE and IHC studies (Fig 4C and 4D). While CDK9 protein expression stayed relatively constant with flavopiridol drug treatment, we noted a decrease in the downstream target MCL1 (Fig 4C). When quantified, there was a decrease in Ki67 expression in the flavopiridol-treated (8.7 ± 1.6%; mean percentage stained ± standard deviation %) compared to the vehicle-treated control (16.9 ± 0.8%) tumors suggesting a decrease in cellular proliferation with drug treatment (*p* = 0.0015) (Fig 4D).

## Discussion

In our study, flavopiridol demonstrated potent inhibition of ATC cell line growth, migration and clonogenic potential *in vitro*. Flavopiridol also inhibited tumor growth in a PDX model demonstrating its potential as a therapeutic agent in ATC. Recent clinical studies have focused on the utilization of small-molecule kinase inhibitors. Such therapies have shown great promise to improve patient outcomes in ATC. The most impressive have been dramatic responses and prolonged survival with the combination of dabrafenib and trametinib in BRAF^V600E^ mutant ATC, which has led to FDA approval [27–29]. Given the observed *in vitro* and *in vivo* activity of flavopiridol, there may be a role in combination with kinase inhibitors or concurrent with radiation. Further studies are needed to elucidate its optimal role prior to clinical trials.

We attempted to uncover the mechanism responsible for the potency of flavopiridol across our ATC cell line panel. Flavopiridol has previously been demonstrated to be both cytostatic and cytotoxic in prostate, esophageal, lung and head and neck carcinoma cell lines [24], which has been reported to be mediated through CDK9 inhibition. Our findings demonstrated that flavopiridol impaired cycle progression as demonstrated by arrest in the G2M phase and downregulated cell cycle proteins CDK9 and MCL1 *in vitro* and MCL1 *in vivo*. However, knockdown of CDK9 was only sufficient to impair growth of one of two cell lines, and knockdown of CDK9 did not affect cell line flavopiridol sensitivity. Taken together, this suggests that CDK9 cannot be the sole mechanism of action of this drug. Interestingly, we have previously observed that knockdown of the primary reported target of a kinase inhibitor does not affect sensitivity to the drug [18], suggesting that off target effects are likely responsible for the antiproliferative effect of some agents. As flavopiridol has been shown to inhibit a number of other CDKs in addition to CDK9 (CDK1, CDK2 and CDK4), it is plausible that the siRNA knockdown of CDK9 alone would not be sufficient to cause resistance as the other targets still exist [10]. Knockdown of other CDKs in combination with CDK9 may aid in elucidating the true anticancer mechanism of flavopiridol. Further investigation is needed to fully understand the chief anticancer mechanism of action of this drug.

In this study, we established a novel PDX model from primary tumor of a patient with stage T4N1M1 disease. The use of PDX models using patient specimens are a highly valuable entity for preclinical drug development because such PDX drug responses have been shown to strongly correlate with drug response of the original tumor, making them highly useful for drug discovery efforts such as our own [30]. Since ATC is so rare, there is a paucity of PDX models that have been reported in the literature. We examined changes in tumor volume over time for all models treated with flavopiridol relative to the vehicle-treated arm and demonstrated that flavopiridol treatment resulted in reduction in both tumor size, weight and protein expression of the marker Ki67 associated with cellular proliferation as indicated by immunohistochemistry studies. A potential limitation in this study lies in the sample size used and as preclinical evaluation of flavopiridol continues, this model can potentially be utilized to test combination treatments and the potential of concurrent treatment with radiation.

## Conclusions

Collectively, our findings identify flavopiridol as a potent small molecule inhibitor that is highly active in both cell lines and a PDX model of ATC. While flavopiridol demonstrated an effect both *in vitro* at nanomolar concentrations and *in vivo*, it is evident that CDK9 inhibition alone is not the only mechanism contributing to this effect and therefore, further work needs to be done to understand the effects of this promising agent. Given the results from this study and the urgent need for therapeutic options for patients facing ATC, further investigations are necessary to improve our understanding of the clinical validity of this drug as an option for patients to delay disease progression and improve overall patient outcomes.

## Supporting information

S1 File The ARRIVE guidelines checklist. (PDF)

S1 Fig Uncropped western blot for Fig 2, panel A. Detection of target proteins was performed using Luminata Forte Western HRP substrate (EMD Millipore, Burlington, MA, USA). α-tubulin was used as a loading control.
(TIF)

S2 Fig Uncropped western blot for Fig 3, panel A. Detection of target proteins was performed using Luminata Forte Western HRP substrate (EMD Millipore, Burlington, MA, USA). α-tubulin was used as a loading control.
(TIF)
