# Supplementary figures and images for: Flavopiridol causes cell cycle inhibition and demonstrates anti-cancer activity in anaplastic thyroid cancer models
Source: PLoS One. 2020 Sep 24;15(9):e0239315. doi: 10.1371/journal.pone.0239315 (PMC7514001; doi:10.1371/journal.pone.0239315)

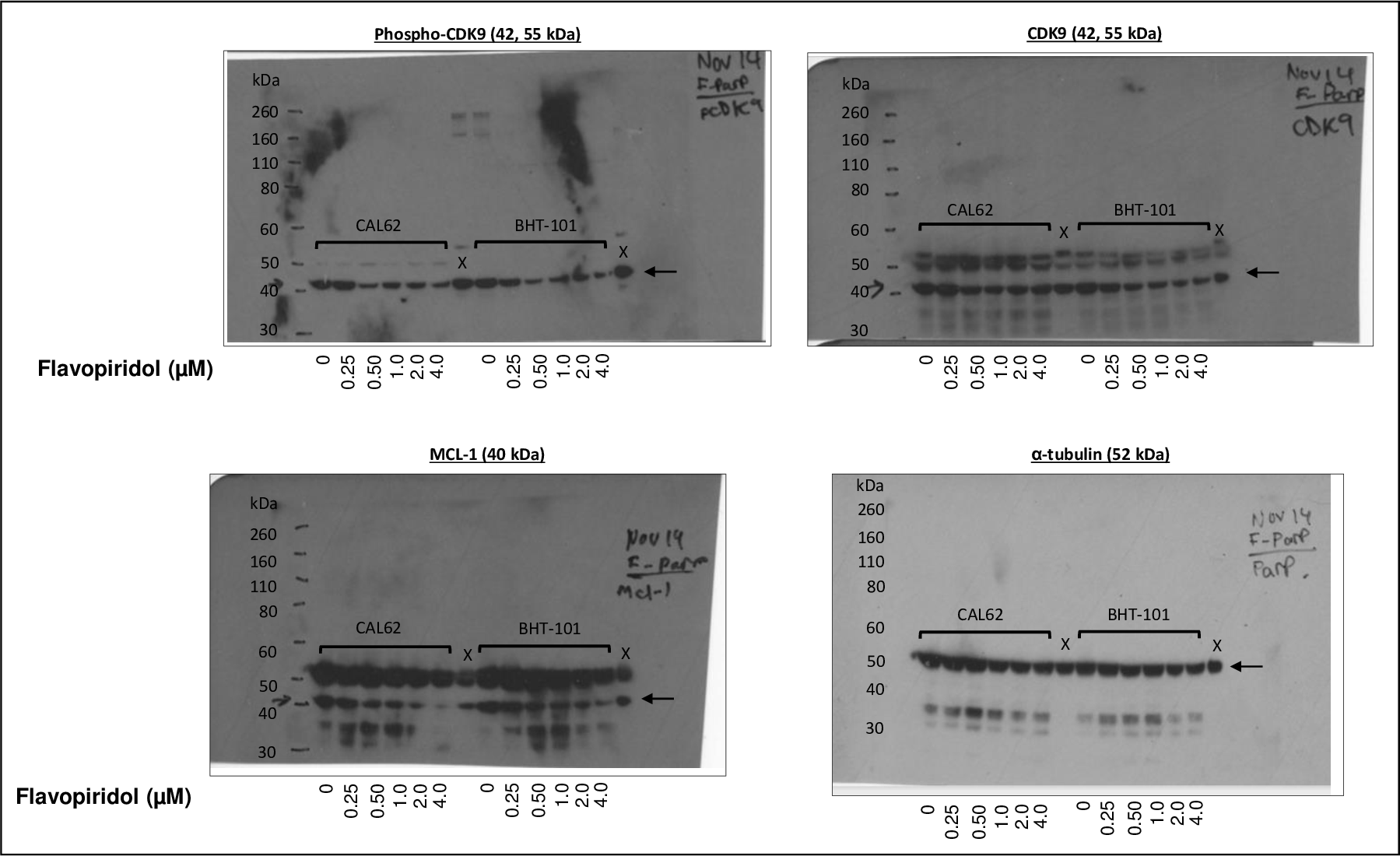

Supplement: S1 Fig — Detection of target proteins was performed using Luminata Forte Western HRP substrate (EMD Millipore, Burlington, MA, USA). α-tubulin was used as a loading control. (TIF) [file pone.0239315.s002.tif]

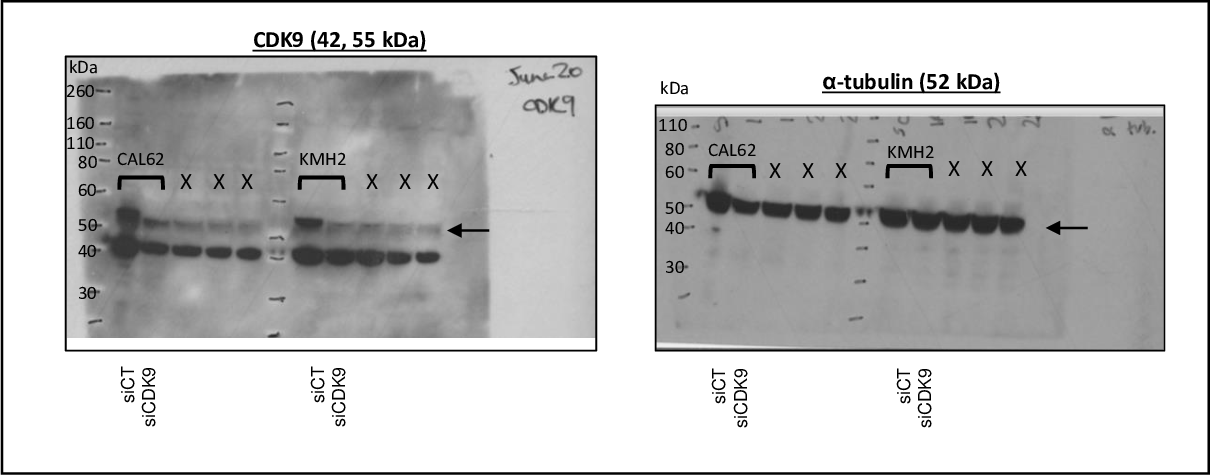

Supplement: S2 Fig — Detection of target proteins was performed using Luminata Forte Western HRP substrate (EMD Millipore, Burlington, MA, USA). α-tubulin was used as a loading control. (TIF) [file pone.0239315.s003.tif]
